# Supplementary material for: The mycobacterium lipid transporter MmpL3 is dimeric in detergent solution, SMALPs and reconstituted nanodiscs
Source: RSC Chem Biol. 2024 Jul 29;5(9):901–13. doi: 10.1039/d4cb00110a (PMC11352979; doi:10.1039/d4cb00110a)
Supplement: CB-005-D4CB00110A-s001 [file CB-005-D4CB00110A-s001.pdf]

Supplementary Information (SI) for RSC Chemical Biology.
This journal is © The Royal Society of Chemistry 2024

Cioccolo et al, Supplementary Information

Supplementary Figure 1

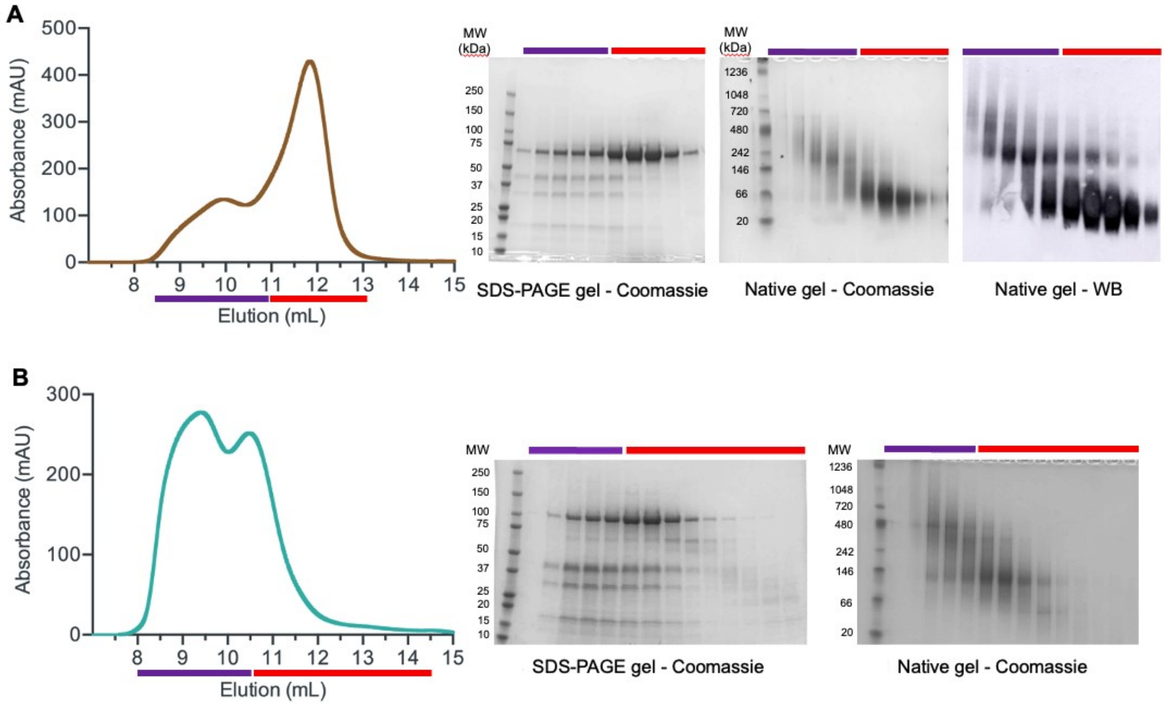

Analysis of *Msmeg* MmpL3 ΔC and MmpL3 FL in SMALPs. A Results for *Msmeg* MmpL3 ΔC into SMALPs: from left to right, SEC profile, Coomassie-stained SDS-PAGE and native gels for early-eluted fractions (purple bar) and late-fractions (red bar). On the right anti-His WB on native gel performed on same fractions. **B** Results for *Msmeg* MmpL3 FL into SMALPs: from left to right, SEC profile, Coomassie-stained SDS-PAGE and native gels for early-eluted fractions (purple bar) and late-fractions (red bar). MW = Molecular weight markers.

Supplementary Figure 2

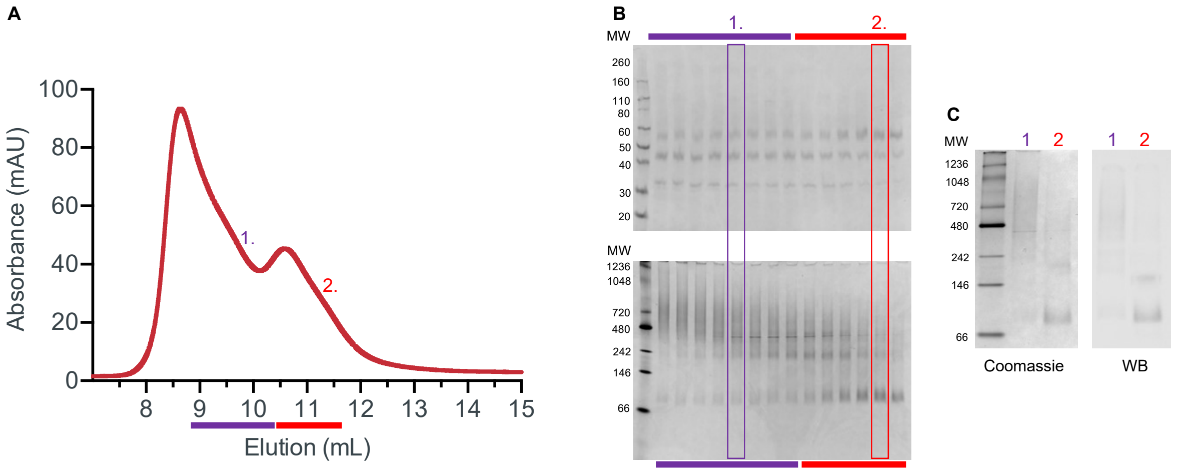

Analysis of *Mtb* MmpL3 ΔC in SMALPs. A SEC profile showing an early-eluting peak (purple bar) with fraction 1, and a late-eluting peak (red bar) with fraction 2. **B** Coomassie-stained SDS-PAGE (top) and native (bottom) gels for early-eluted fractions (purple bar) and late fractions (red bar). Bands for fraction 1 and 2 are highlighted in purple and red boxes, respectively. **C** Coomassie-stained native gel (left) performed on same fractions and anti-His WB (right). MW = Molecular weight markers.

Supplementary Figure 3

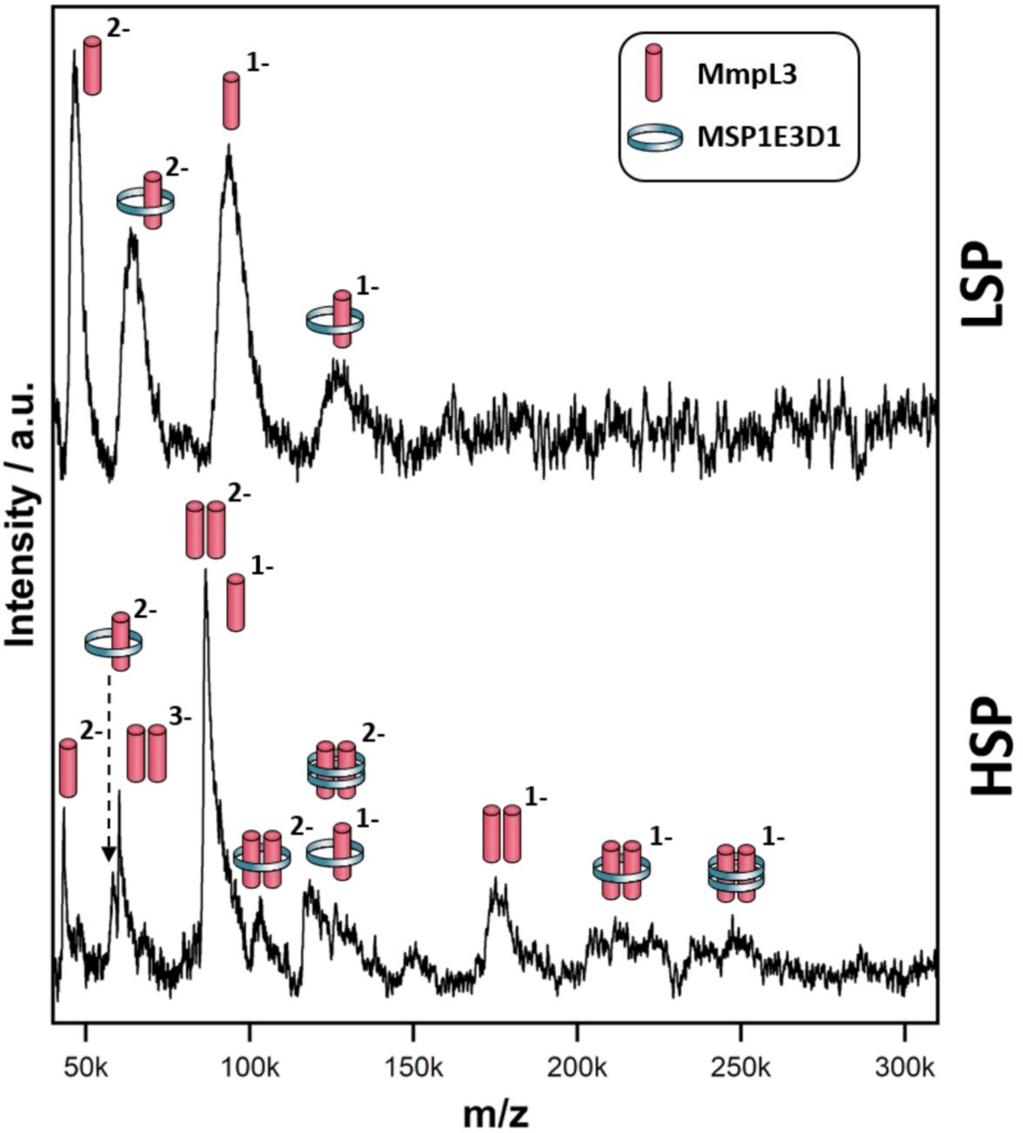

LILBID analysis of *Msmeg* MmpL3 DC protein in MSP-NDs at harsh laser intensity. nMS spectra of the *Msmeg* MmpL3 DC protein in MSP-NDs separating at low (upper panel) and high (lower panel) sucrose percentage (LSP) measured at23 mJ laser intensity. The different protein-MSP combinations detected in each sample and relative charged species are indicated with pictograms. Importantly even though the higher laser intensity causes loss of the associated MSP in most cases, it does not cause complete loss of MmpL3 dimer.

Supplementary Figure 4

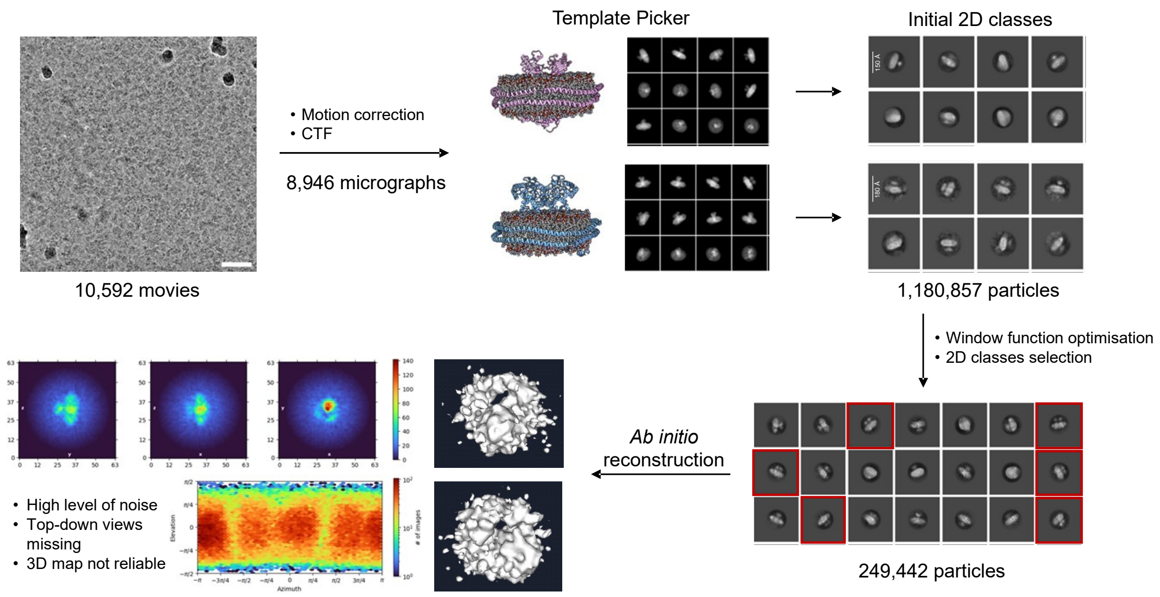

Cryo-EM workflow. Movies were pre-processed and micrographs extracted, following motion correction and CTF. synthetic models of NDs carrying either monomeric or dimeric MmpL3 were used as template to pick particles and obtain initial 2D classes. Percentage of box size was optimized to adjust focus on single particles and perform several rounds of 2D classifications. Preliminary *ab initio* reconstruction was attempted but high level of noise and lack of top-down views gave 3D maps considered not reliable.

Supplementary Figure 5

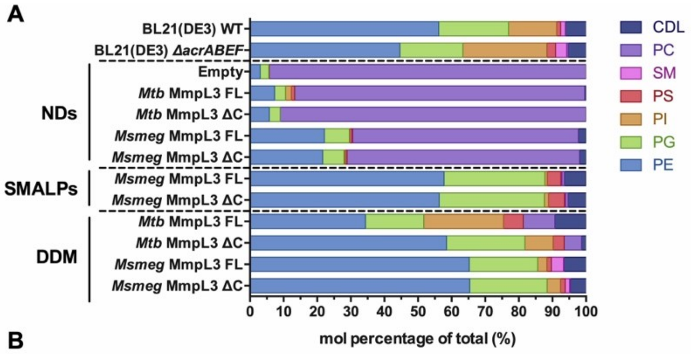

B

Lipid species	MS/MS	Membrane (Δ*acrABEF*)	*Msmeg* MmpL3 ΔC		
			DDM	NDs	SMALPs
PE 30:0	14:0/16:1	3.84	6.48	1.31	3.38
**PE 32:1**	**16:1/16:0 or 14:0/18:1**	**8.63**	**11.82**	**4.02**	**12.03**
**PE 33:1**	**16:0/17:1**	**7.02**	**11.99**	**5.43**	**12.17**
PE 33:2	16:1/17:1	0.79	3.38	0.67	3.22
**PE 34:1**	**16:0/18:1**	**8.27**	**9.95**	**4.29**	**7.32**
PE 34:2	18:1/16:1	1.84	2.69	1.01	3.31
PE 35:1	16:0/19:1	2.31	2.88	0.40	1.51
PE 35:2	18:1/17:1	1.18	2.02	1.07	2.45
PE 36:2	18:1/18:1	1.50	2.11	0.95	1.97
PE O;38:6	/	3.40	3.62	0.82	4.85
**PG 32:1**	**16:1/16:0 or 14:0/18:1**	**3.80**	**4.90**	**1.85**	**8.50**
**PG 33:1**	**16:0/17:1**	**2.42**	**2.84**	**0.75**	**4.61**
**PG 34:1**	**16:0/18:1**	**4.15**	**4.82**	**1.81**	**7.78**
PC 34:1	16:0/18:1	0.23	/	69.06	/
**Abundance species >2%**		60.0%*	69.5%	82.8%	71.8%
**n. of species >2%**		13*	13	4	12
**Total n. of lipid species**		109	99	59	70
**Total n_{lipids} (nmol)**		4.65	4.49	2.52	1.54

* including 4x PI species not included in table.

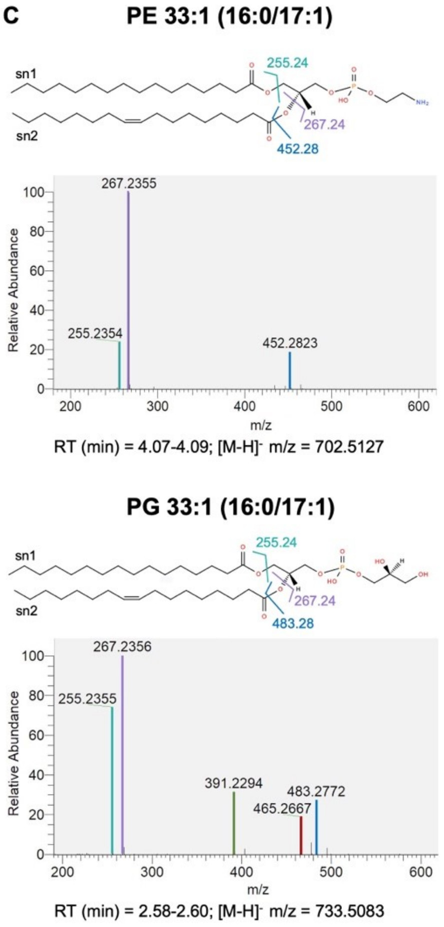

Lipidomics analysis of MmpL3 constructs. Proteins were isolated in DDM, SMALPs or isolated in DDM and then reconstituted into NDs prior to lipidomics analysis. Lipidomics analysis of BL21(DE3) WT and Δ*acrABEF* cell strains showed AcrB knock out had no effect on lipid profile. **A.** Main lipid species identified in MmpL3 samples, empty NDs and *E. coli* membranes, selecting a 2 - 7 min range as retention time on chromatogram. For each sample, abundance of lipid species was calculated as relative mol % over total lipid moles and was plotted as stacked bars, with individual lipids coloured according to the key. **B.** Abundance of main lipid species in *Msmeg* MmpL3 ΔC in DDM, POPC NDs and SMALPs. Table shows relative abundance of lipid species as mol % of total lipids per sample (bottom row) identified in MmpL3 and *E. coli* BL21(DE3) Δ*acrABEF* membranes, used as reference. MS/MS column indicates acyl chains identified using fragmentation protocol. **C.** MS/MS fragmentation spectra for PE 33:1 (top panel) and PG 33:1 (bottom panel) lipid species. Main m/z values for acyl chains fragments were identified for both species. A m/z = 255.24 indicated a sn1 $RCOO^-$ ion, while m/z = 267.24 indicated a sn2 $RCOO^-$ ion. Loss of sn2 as ketene from $[M-H]^-$ gave m/z values of 452.28 and 483.28 for PE and PG, respectively. Other fragments identified for PG 33:1 are shown together with their relative m/z values.

Species-specific retention times and relative $[M\text{-}H]^-$ m/z values are also reported below spectra.

Supplementary Figure 6

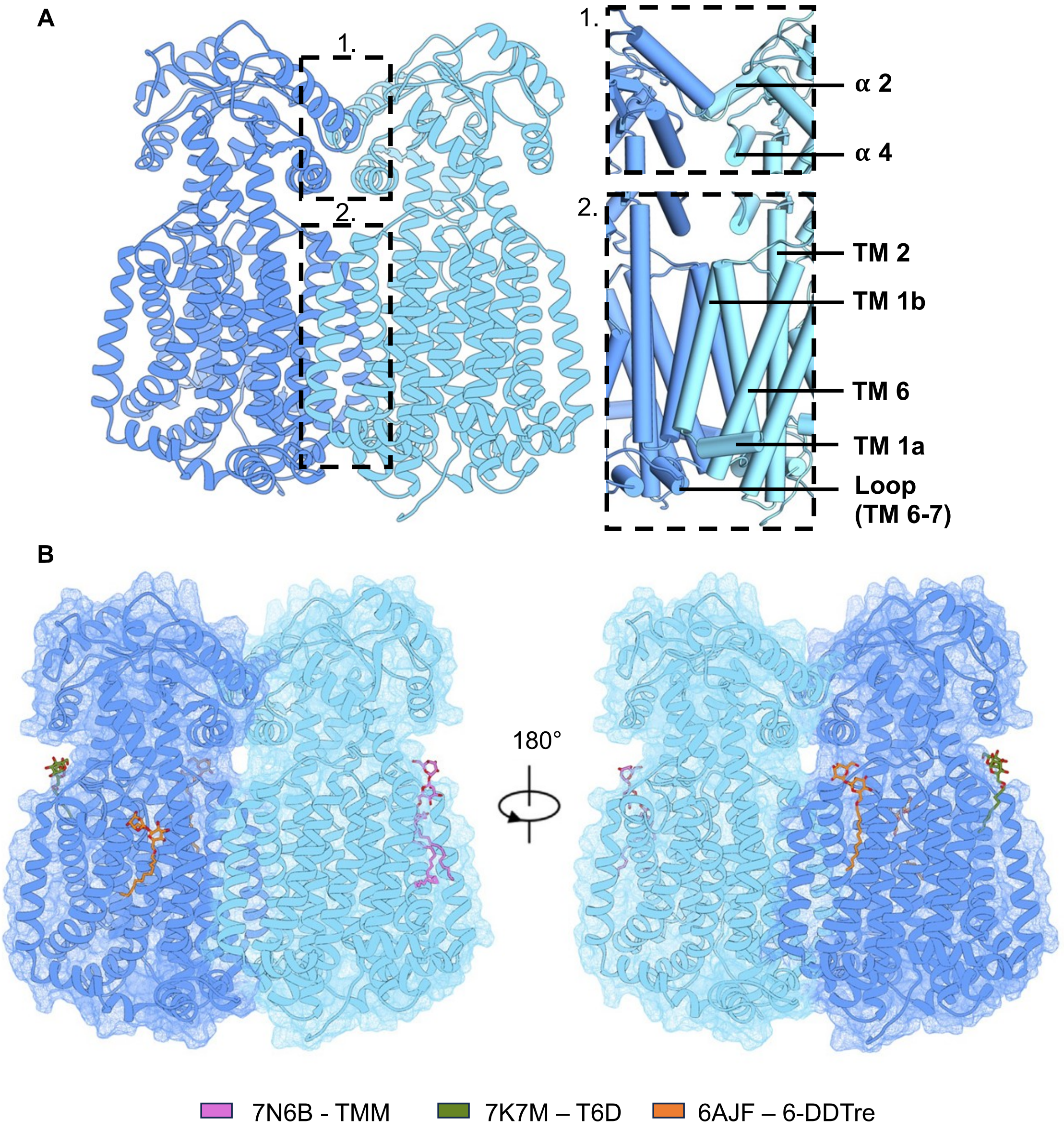

The interaction interface of the MmpL3 dimer. A *Msmeg* MmpL3 dimer model generated using GalaxyHomomer server starting from PDB 7N6B (left hand panel). The individual protomers are coloured dark and pale blue with the helices shown in ribbon representation. The regions highlighted in the dashed boxes indicate those parts of the protein (both TM domains and PD regions) involved in the predicted dimer interface. A zoomed in image of the relevant secondary structure regions involved in the predicted dimer interface shown in cylinder representation (right hand panel). **B** *Msmeg* MmpL3 dimer model with bound ligands. Surface representation of the dimer model. MmpL3 structure was superimposed to 7N6B, 7K7M and 6AJF. Co-purified ligands from the reference PDBs and their binding sites are shown: trehalose monomycolate (TMM, pink), trehalose 6-decanoate (T6D, green) and lauryl-6-rehaloside (6-DDTre, orange).

Supplementary Figure 7

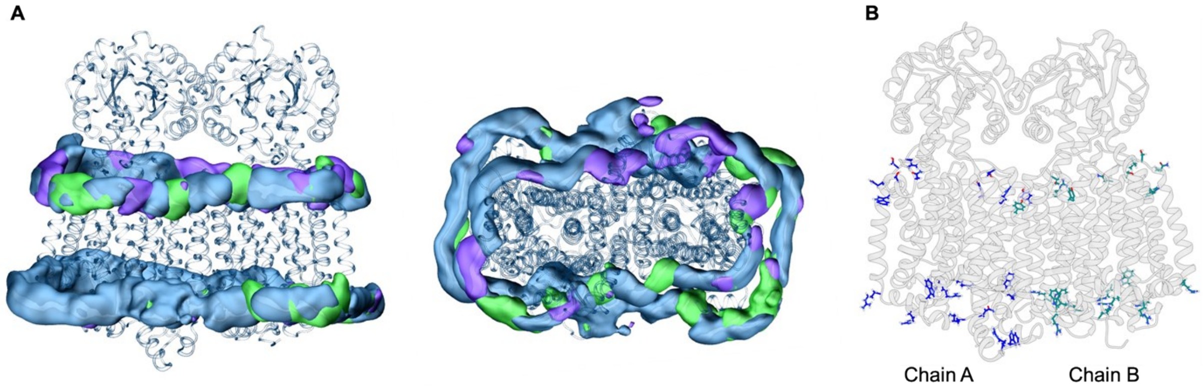

Protein-lipid interactions of the *Msmeg* MmpL3 dimer. *Msmeg* MmpL3 dimer: protein-lipid interactions. **A** Front (left) and top (right) view of averaged lipid occupancy map generated in VMD using a selection of PE (blue), PC (purple) and PG (green) molecules within 6 Å from protein along the whole trajectory. **B** Main protein residues per chain involved in hydrogen bond formation with lipid molecules on top and bottom leaflets (front view).

Supplementary Figure 8

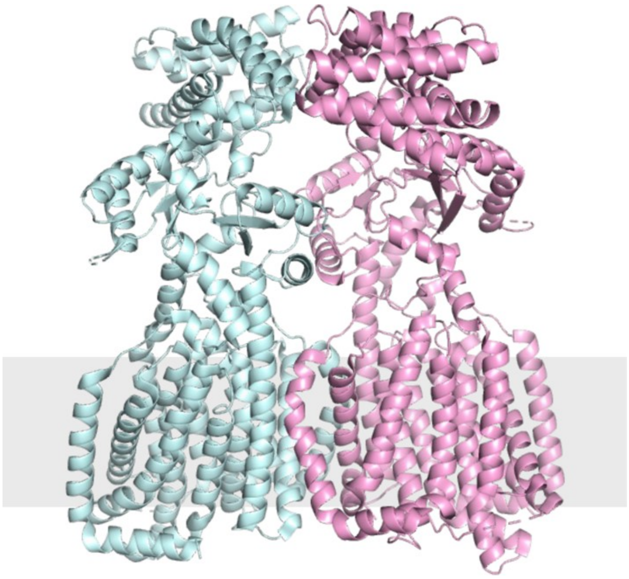

Structure of the HpnN dimer (PDB 5KHN). The individual protomers are shown in cyan and pale pink respectively and the grey rectangle indicates the approximate position of the membrane. It is clear that the interactions involved in dimer formation involve both the transmembrane and periplasmic domains.
